# Supplementary material for: The CaMKII/MLC1 Axis Confers Ca2+-Dependence to Volume-Regulated Anion Channels (VRAC) in Astrocytes
Source: Cells. 2022 Aug 26;11(17):2656. doi: 10.3390/cells11172656 (PMC9454758; doi:10.3390/cells11172656)
Supplement: Supplementary file 1 [file cells-11-02656-s001.zip › cells-1830475-supplementary.pdf]

Supplementary information

The CaMKII/MLC1 axis confers Ca^{2+}-dependence to volume-regulated anion channels (VRAC) in astrocytes

Maria Stefania Brignone [1 †], Angela Lanciotti [1 †], Antonio Michelucci [2 †], Cinzia Mallozzi [1], Serena Camerini [3], Luigi Catacuzzeno [2], Luigi Sforna [2], Martino Caramia [2], Maria Cristina D'Adamo [4], Marina Ceccarini [5], Paola Molinari [6], Pompeo Macioce [1], Gianfranco Macchia [3], Tamara Corinna Petrucci [1], Mauro Pessia [7,8], Sergio Visentin [6] and Elena Ambrosini [1,*]

[1] Dept. of Neuroscience, Istituto Superiore di Sanità, 00169 Rome, Italy.
[2] Dept. of Chemistry, Biology and Biotechnology, University of Perugia, Italy.
[3] Core Facilities (FAST), Istituto Superiore di Sanità, 00169 Rome, Italy.
[4] Dept. of Medicine and Surgery, LUM Giuseppe Degennaro University, Bari, Italy.
[5] National Centre for Rare Diseases, Istituto Superiore di Sanità, 00169 Rome, Italy.
[6] National Centre for Drug Research and Evaluation (FARVA), Istituto Superiore di Sanità, 00169 Rome, Italy.
[7] Dept. of Physiology and Biochemistry, Faculty of Medicine and Surgery, University of Malta, Malta;
[8] Dept. of Physiology, College of Medicine and Health Sciences, United Arab Emirates University, Al Ain, United Arab Emirates.

* Correspondence: elena.ambrosini@iss.it; Tel.:+390649902037

† These authors equally contributed to this work

Figure S1

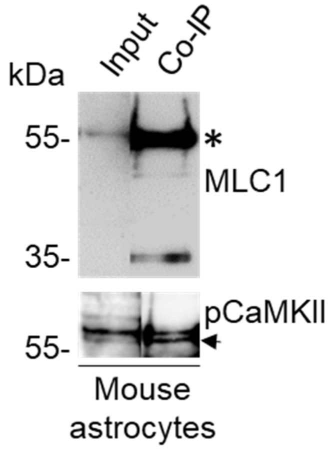

Figure S1. *Phospho (p)CaMKII interacts with MLC1 protein in primary mouse astrocytes.* Immunoprecipitation (IP) of MLC1 protein from primary mouse astrocytes with anti-pCaMKII pAb. Immunoblottings performed with the anti-MLC1 pAb (asterisk) and anti-CaMKII pAb (arrow) shows MLC1/CaMKII co-IP.

Figure S2.

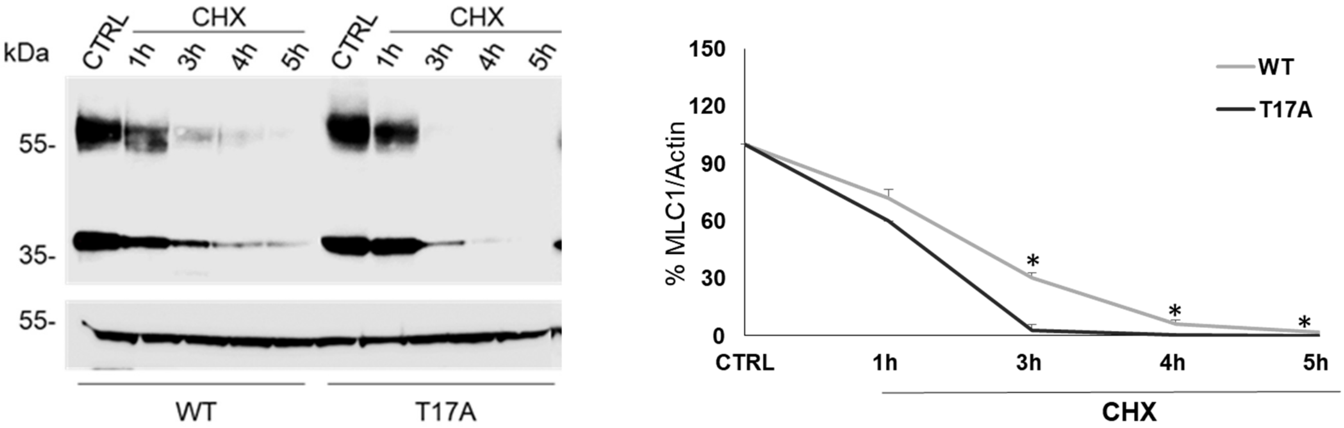

Figure S2. *MLC1-T17A mutant protein shows a reduced stability when compared to the MLC1-WT protein.*

WB analysis of U251 cells expressing MLC1-WT, and the T17A mutant, untreated (CTRL) or treated with cycloheximide (CHX, 100 µg/mL) for 1, 3, 4 and 5 h reveals a decrease of the T17A mutant protein stability when compared to MLC1-WT. The graph indicating the densitometry analysis of MLC1 protein bands normalized with the amount of actin is shown. Data are expressed as percentage of the value measured in control untreated cells (means ± SEM of 3 replicates for each type of experiments; *p< 0.05 calculated using non-parametric test).

Figure S3

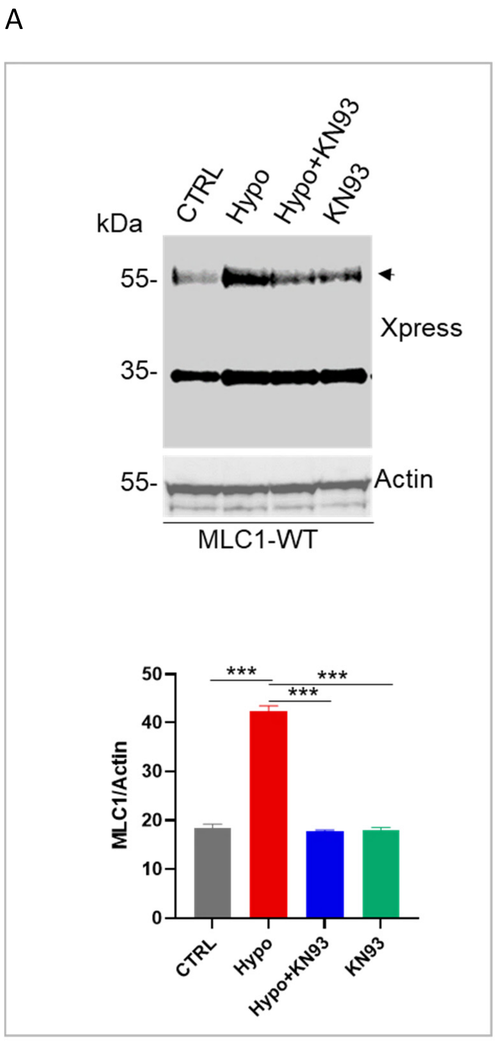

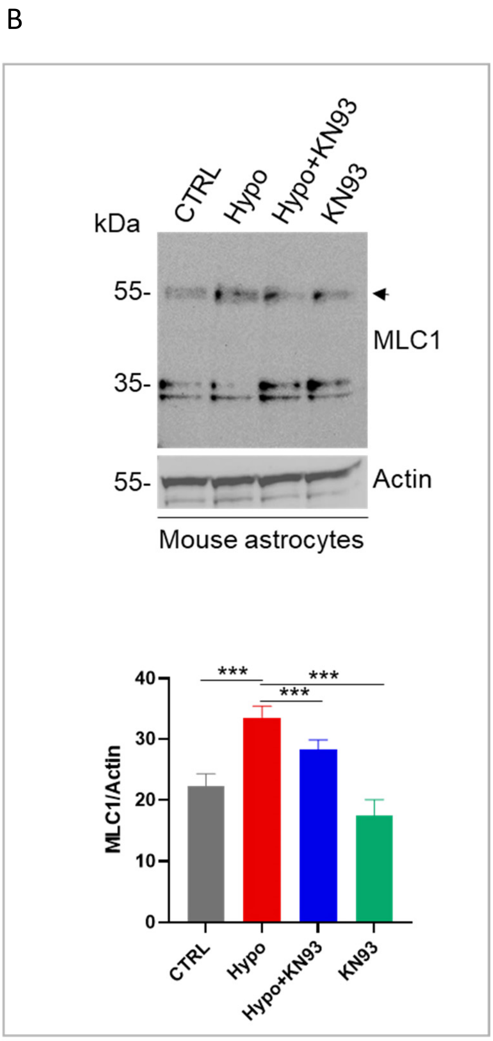

Figure S3. *CaMKII inhibition reduces MLC1 protein dimerization in U251 cells and primary mouse astrocytes.*

WB analysis of U251 cell expressing MLC1-WT (A) and primary mouse astrocytes (B) shows the increase of the dimeric form of MLC1 (arrow) following a 15 min treatment with hyposmotic solution (Hypo) and its reduction after cell co-treatment with KN93 (Hypo+KN93). Actin is used as a loading control. MW markers are indicated on the left (kDa). The bar graph below the WB represents the densitometry analysis of the MLC1 protein bands normalized with the amount of actin in the corresponding samples. The means $\pm$ SEM of 3 independent experiments are shown. Statistical differences were calculated using non-parametric tests (***$p < 0.0001$)

Figure S4

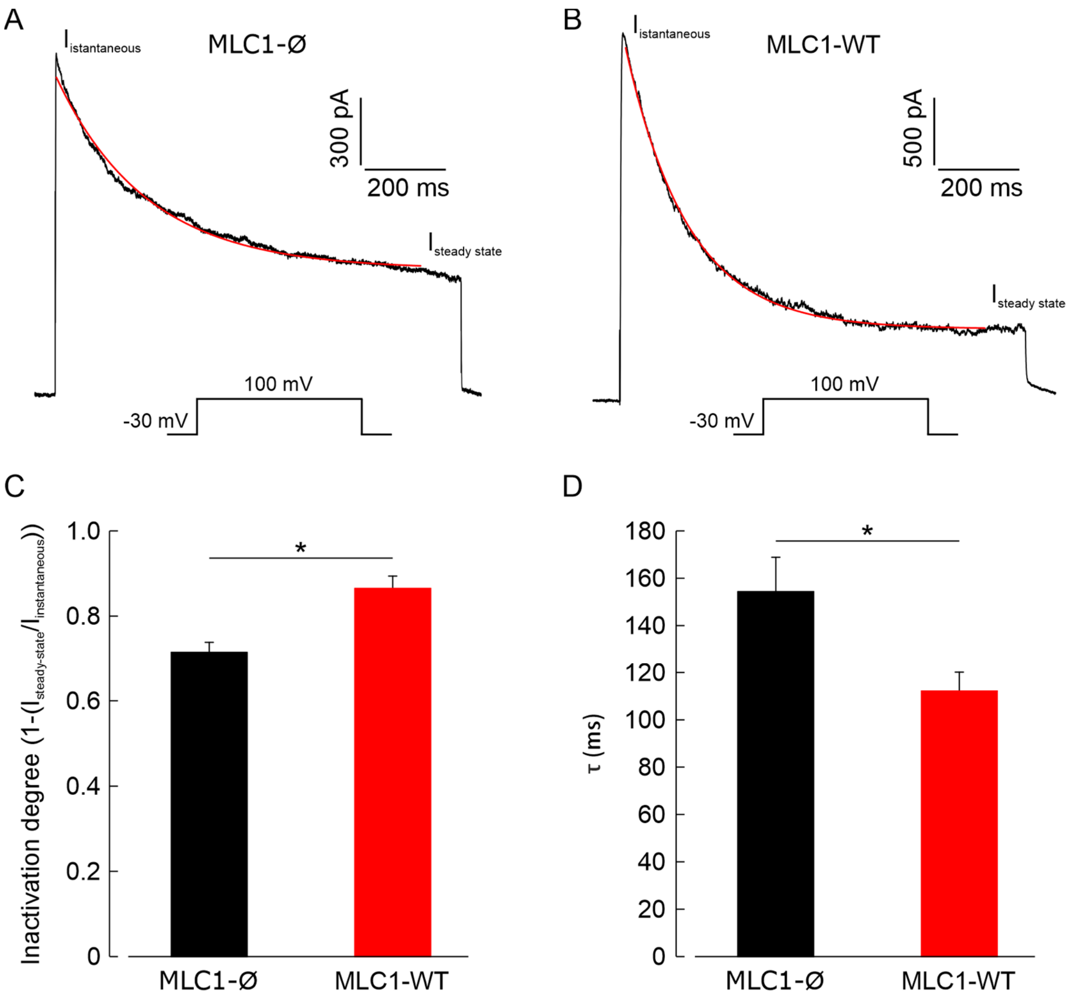

Figure S4. *Effects of MLC1 WT on the degree and kinetics of inactivation of the hypotonic-induced* $I_{Cl,swell}$ *in U251 cells.*

(A, B) Representative current traces evoked by applying 1 s voltage step at 100 mV, from a holding potential of -40 mV in the presence of a 30% hypotonic solution in U251 cells not expressing MLC1 (MLC1-ø, n=5, A) and in MLC1-WT cells, (n=5, B). The red lines in each trace are the monoexponential fits of the current traces, used for the evaluation of the time constant (τ) of current inactivation. (C, D) Bar plots showing the quantitative analysis of the average degree of inactivation (C), calculated as 1-($I_{steady-state}$/$I_{istantaneous}$), where $I_{istantaneous}$ is the current at the peak, and the average values of the time constant resulting from the monoexponential fit of the current decay (D). Data are shown as mean ± SEM (*p<0.05).

Figure S5

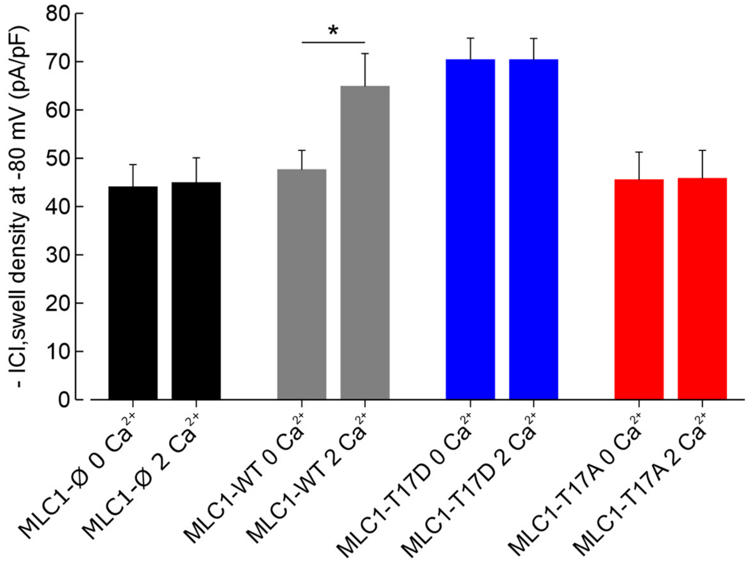

Figure S5. *$I_{Cl,swell}$ density measured in presence and absence of extracellular Ca^{2+} in U251 cell populations.*

Bar plot showing the average current density measured at -80 mV during exposure to 30% hypotonic solution in the presence or absence of external Ca^{2+} in U251 cells not expressing MLC1 (MLC1-Ø, n=7), expressing WT MLC1 (MLC1-WT, n=7) or MLC1 mutants (MLC1-T17D, n=7; and MLC1-T17A, n=7). Data are shown as mean ± SEM (*p<0.05).
